# The complete chloroplast genome of *Akebia trifoliata* subsp. *australis* (Lardizabalaceae), a medicinal plant in China

**DOI:** 10.1080/23802359.2020.1820398

**Published:** 2020-09-16

**Authors:** Aqiao Yu, Qi Tan, Jing Chen, Hui Huang

**Affiliations:** a Key Laboratory of Research and Utilization of Ethnomedicinal Plant Resources of Hunan Province, College of Biological and Food Engineering, Huaihua University, Huaihua, China; b Kunming Institute of Botany, Chinese Academy of Sciences, Kunming, China

**Keywords:** Plastid genome, *Akebia trifoliata* subsp. australis, phylogenetic analysis

## Abstract

*Akebia trifoliata* subsp. *australis* (Diels) T.Shimizu is a medicinal plant in China. Here, the complete chloroplast (cp) genome sequence of *A. trifoliata* subsp. *australis* was assembled and characterized as a resource for future genetic studies. The whole cp genome was 157,952 bp in length, containing of a large single-copy (LSC) region of 86,596 bp, a small single-copy (SSC) region of 19,060 bp, and two inverted repeat (IR) regions of 26,148 bp. The new sequence possessed total 113 unique genes, including 79 protein-coding genes, 30 tRNA genes and 4 rRNA genes. The nucleotide composition was asymmetric (30.3% A, 19.7% C, 19.0% G and 31.0% T) with an overall GC content of 38.7%. The maximum likelihood phylogenetic analysis based on 10 cp genomes indicated that *A. trifoliata* subsp. *australis* was closely related to *Akebia trifoliata* subsp. *trifoliata.* However, *Akebia quinata* was closely related to *Stauntonia obovatifoliola*.

*Akebia trifoliata* (Thumb.) Koidz. subsp. *australis* is a perennial woody plant, belongs to the genus *Akebia* (Lardizabalaceae) and mainly distributed in the eastern part of Asia (Liu et al. 2007). *Akebia trifoliata* subsp. *australis* as well as *A. trifoliata* subsp. *trifoliata* and *A. quinata* that are two other members in genus *Akebia,* have been listed in the Chinese Pharmacopeia (Chinese Pharmacopoeia Commission 2015) and used as traditional herbal medicine over 2000 years. The complete chloroplast (cp) genome of *A. trifoliata* subsp. *trifoliata* and *A. quinata* had been reported (Hong et al. 2020; Min and Tao 2020). However, the complete chloroplast genome of *A. trifoliata* subsp. *australis* is lacking. In this study, the complete cp genome of *A. trifoliata* subsp. *australis* was sequenced, annotated and analyzed by using Illumina high-throughput sequencing platform, which will facilitate the elucidation of phylogenetic evolutionary aspects in the cp genome-wide level in *A. trifoliata* subsp. *australis* and its closely related species in Lardizabalaceae.

The fresh green leaves of *A. trifoliata* subsp. *australis* were collected from Huaihua, Hunan Province, China (N27°33′17.95ʺ, E109°59′54.70ʺ). The corresponding voucher herbarium specimen was stored at the college of biological and food engineering of Huaihua University (No. MT20190501). Total genomic DNA including nuclear and organelle genome was extracted using CTAB protocol. The qualified DNA was used to construct a 150 bp paired-end library for sequencing, using HiSeq2000 platform (Illumina, USA). After sequencing, FastQC (Andrews 2015) and Trimmomatic (Bolger et al. 2014) were used to quality control, and remove low quality sequences and contaminants, respectively. The chloroplast geome was *de novo* assembled using GetOrganelle (Jin et al. 2018). The cp genome sequence was annotated using GeSeq (Tillich et al. 2017). The cp genome sequence of *A. trifoliata* subsp. *australis* had been submitted to NCBI under accession number MT876408.

The complete cp genome sequence of *A. trifoliata* subsp. *australis* has a total of 1,57,952 bp in size with overall GC content 38.7%. The cp genome has a typical quadripartite structure and contains a large single-copy region (LSC) of 86,596 bp, a small single-copy region (SSC) of 19,060 bp, and two inverted repeat regions (IRA and IRB) of 26,148 bp. There were a total of 113 unique genes, including 79 protein-coding genes, 30 tRNA genes and 4 rRNA genes, in *A. trifoliata* subsp. *australis* cp genome. Among these genes, ten protein-coding genes (*atpF*, *ndhA*, *ndhB*, *petB*, *petD*, *rpl16*, *rpl2*, *rpoC1*, *rps12* and *rps16*) and six tRNA genes (*trnA-UGC*, *trnG-UCC*, *trnI-GAU*, *trnK-UUU*, *trnL-UAA* and *trnV-UAC*) contained a single intron, and two protein-coding genes (*clpP* and *ycf3*) possessed two introns.

To understand the phylogenetic position of *A. trifoliata* subsp. *australis* within the genus *Akebia* and the family Lardizabalaceae, we downloaded the complete cp genomes of seven species in Lardizabalaceae and two species in Ranunculaceae. The sequences were aligned using MAFFT v7.307 (Katoh and Standley 2013), and RAxML (Stamatakis 2014) was used to construct a maximum likelihood (ML) tree with *Megaleranthis saniculifolia* and *Aquilegia coerulea* as outgroups. The ML phylogenetic tree was inferred with strong support and used the bootstrap values from 1000 replicates at all the nodes. The phylogenetic tree showed that *A. trifoliata* subsp. *australis* was closely related to *A. trifoliata* subsp. *trifoliata* (Figure 1). However, *A. quinata* was closely related to *Stauntonia obovatifoliola*. This published *A. trifoliata* subsp. *australis* cp genome will provide useful information for phyogenetic and evolutionary studies in Lardizabalaceae.

**Figure 1.**
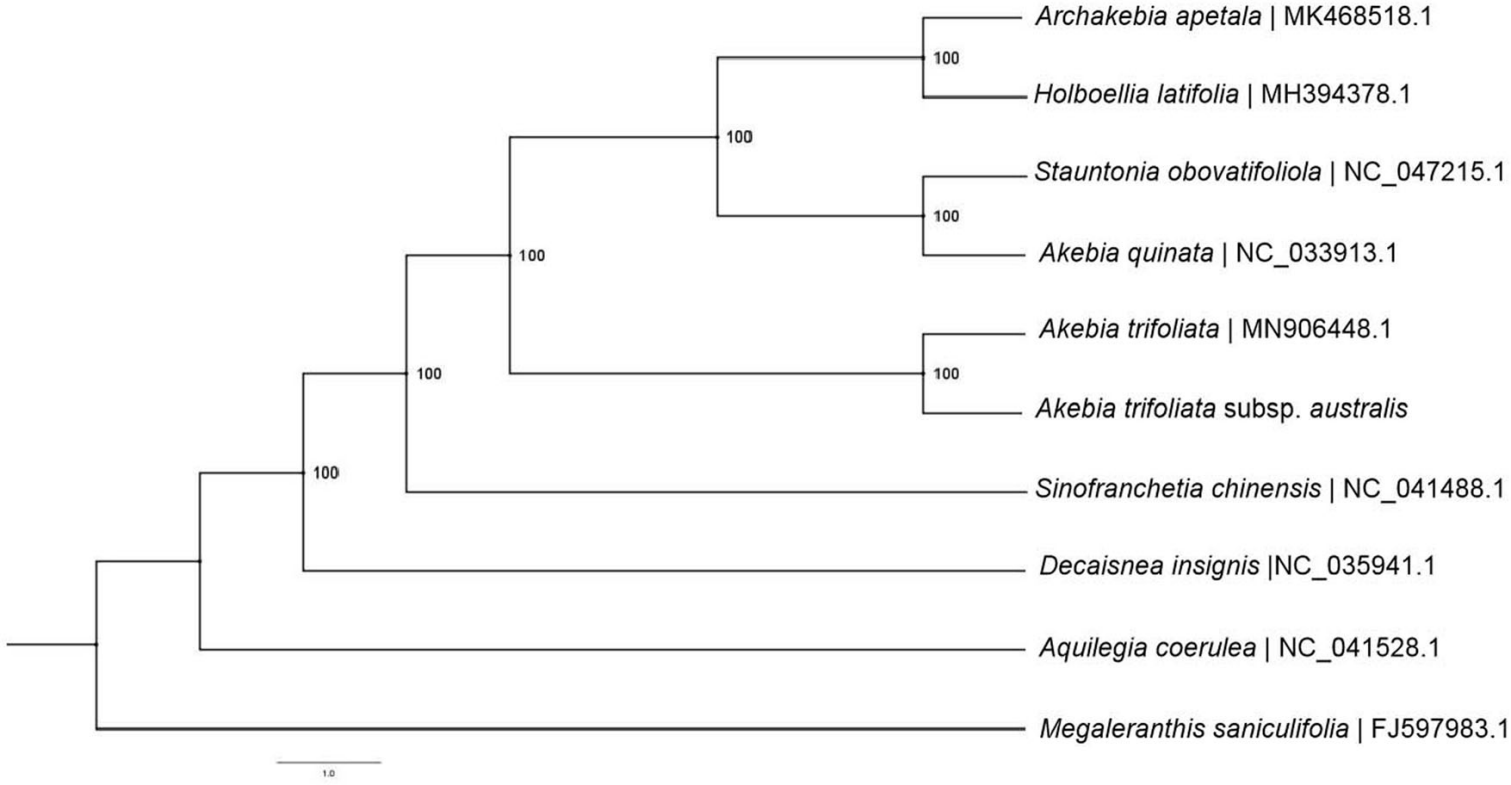
The phylogenetic ML tree based on the complete chloroplast sequence of 10 species. ML bootstrap support value presented at each node. The accession numbers are in the figure.

## Data Availability

The data that newly generated in this study is deposited in NCBI (www.ncbi.nlm.nih.gov), with accession number MT876408.
